# Outstanding Tensile Properties and Their Origins in Twinning-Induced Plasticity (TWIP) Steels with Gradient Substructures

**DOI:** 10.3390/ma13051184

**Published:** 2020-03-06

**Authors:** Huihui Zhi, Cheng Zhang, Zihui Guo, Stoichko Antonov, Yanjing Su

**Affiliations:** 1 Beijing Advanced Innovation Center for Materials Genome Engineering, University of Science and Technology Beijing, Beijing 100083, China; 2 Corrosion and Protection Center, University of Science and Technology Beijing, Beijing 100083, China; 3 Department of Microstructure Physics and Alloy Design, Max-Planck-Institut für Eisenforschung, 40237 Düsseldorf, Germany

**Keywords:** twinning-induced plasticity (TWIP) steels, strength, ductility, gradient substructures

## Abstract

The low yield strength (~300 MPa) of twinning-induced plasticity (TWIP) steels greatly limits their structural applications in the industrial field. Conventional strengthening mechanisms usually cause an enhancement of yield strength but also a severe loss of ductility. In this research, gradient substructures were introduced in the Fe-22Mn-0.6C TWIP steels by different pre-torsional deformation in order to overcome the above limitations. The substructure evolution, mechanical properties, and their origins in gradient-substructured (GS) TWIP steels were measured and compared by electron backscattered diffraction (EBSD), monotonous and loading-unloading-reloading (LUR) tensile tests. It was found that a simple torsional treatment could prepare gradient twins and dislocations in coarse-grained TWIP steel samples depending on torsional strain. The uniaxial tensile tests indicated that a superior combination of high yield strength, high ultimate strength, and considerable ductility was simultaneously obtained in the GS samples. The high yield strength and high ultimate tensile strength were attributed to synergetic strengthening mechanisms, viz., dislocation strengthening, due to the accumulation of high density of dislocations, and very high back stress strengthening due to gradient substructure distribution, which was accommodated through pile-ups of extra geometrically necessary dislocations (GNDs) across the sample-scale. Additionally, high ductility originated from gradient substructure-induced back stress hardening. The present study is also beneficial to the design efforts of high strength and high ductility of other heterogeneous-structured TWIP alloy systems.

## 1. Introduction

High manganese twinning-induced plasticity (TWIP) steels have attracted great attention from the automotive industry in recent years due to their outstanding combination of mechanical properties, viz., high ultimate tensile strength (~800 MPa), high ductility (~80%), and enhanced strain hardening capability [1,2]. However, one key factor limiting the structural applications of TWIP steels is their low yield strength, which is about 300 MPa, arising from their single-phase microstructure, where the sole strengthening mechanism is dislocation strengthening during the initial deformation stages [3]. The high yield strength is particularly important for avoiding overload conditions, while high ductility is important for absorbing the impact energy, which the automobile may be subjected to [1]. Up to now, tremendous research efforts have been made to improve the mechanical properties of TWIP steels by precipitation strengthening [4], solid-solution strengthening [5], dislocation strengthening [6], etc. These conventional strengthening mechanisms, based on the concept of a homogenous microstructure design, lead to either an insufficient enhancement of yield strength or a great loss of the material’s ductility by sacrificing the strain hardenability.

Recent studies have shown that a heterogeneous microstructure design is an effective way to simultaneously strengthen and toughen materials and has been applied to various alloy systems, i.e., Ti alloys [7], brass [8], and Fe alloys [9,10]. The heterogeneous microstructure can be categorized into a multi-modal grain size microstructure, heterogeneous lamella structure, nano-twinned structure, gradient structure, etc. Among these categories, the gradient structure, i.e., gradient dislocation density, phase content, or grain size distribution, can be produced by a surface mechanical attrition treatment (SMAT) [11] and surface mechanical grinding treatment (SMGT) [12]. For TWIP steels, particularly in a Fe-10Mn-3Ni-0.6C TWIP thin steel sample, a good combination of high strength and ductility was obtained through the formation of a 50 µm thick gradient substructure produced through the SMAT technique [11]. However, the limited depth of the gradient structure (about a few hundred microns) formed by the general processing ways hinders the applicability to large-scale components. Recently, pre-torsion deformation, which can introduce gradient microstructures at larger scales, has been used to obtain good mechanical properties of Cu [13], Mg [14], and steels [15]. Using the same method, Wei et al. [16] found that a synergy of strength and ductility can be achieved through gradient nanotwins in a dumbbell-shaped TWIP steel specimen. They explained this phenomenon using an activation of different twinning systems along the depth of the steel sample based on finite element (FE) simulation investigations. Zhao et al. [17] also studied the excellent mechanical properties of gradient nanograined materials using a strain gradient plasticity model. In their numerical model, they discussed the effects of grain size, geometrically necessary dislocations (GNDs), and back stress on tensile response. However, to the authors’ knowledge, experimental characterizations of the substructure evolution, i.e., deformation twinning and GND densities, and a detailed quantification of their respective roles in strengthening and strain hardening of gradient-substructured (GS) TWIP steels during tensile deformation, are still rare. Therefore, this study aims to clarify the relationships among the sample processing, resulting microstructures, and mechanical properties of a GS TWIP steel in detail. We believe that the present study is also beneficial to the design efforts for high strength and high ductility in other heterogeneous-structured TWIP alloy systems.

## 2. Materials and Methods

### 2.1. Material and Sample Preparation

In the present study, an ingot of Fe-22Mn-0.6C TWIP steel with measured compositions of 21.56 wt.% Mn, 0.56 wt.% C, and balanced Fe was prepared by vacuum induction melting. To alleviate Mn segregation, the ingot was homogenized at 1150 °C for 2 h in a protective argon gas atmosphere. Subsequently, hot-rolling was performed at temperatures between 1100 °C and 950 °C to form a square plate with dimensions of 50 mm × 50 mm. Finally, the plate was solution annealed at 1050 °C to ensure microstructural homogeneities.

Dumbbell-shaped samples with a gauge section of 4 mm in diameter and 28 mm in length were excised from the abovementioned plate. This sample condition was termed as coarse-grained. Subsequently, pre-torsion to 90°, 180°, and 360° was applied on the above coarse-grained samples using an MTS 809 axial/torsional testing machine to fabricate GS samples of different degrees. The details of the processing process can be found in Section 3.1.

### 2.2. Mechanical Tests

Quasi-static, monotonous, and loading-unloading-reloading (LUR) tensile tests were performed on the coarse-grained and GS samples at a strain rate of 1 × 10^−4^ s^−1^ at room temperature using an MTS Landmark tensile testing machine. An extensometer was used to measure the strain during tensile testing. The resolutions of the stress and strain measurements were 1 MPa and 1.0 × 10^−5^, respectively. All tensile tests were repeated at least three times to ensure the reliability and reproducibility of the data.

### 2.3. Substructural Characterizations

#### 2.3.1. Electron Backscattered Diffraction (EBSD) Characterizations

The microstructural analysis of the samples was performed using Zeiss Gemini SEM 500 equipped with a fully automatic Oxford Instruments Aztec 4.0 EBSD system (Oxford, UK) and a complementary metal-oxide semiconductor (CMOS)-based Symmetry EBSD detector (Oxford, UK). The following settings were used during EBSD data acquisition: 70° sample tilt, 20 kV accelerating voltage, 15 mm working distance, 60 μm aperture size, 200 nm step size, and 1 × 1 binning, which provide extremely high spatial and angular resolutions under the measuring limit of the EBSD instrument. The present EBSD parameters provide a resolution of ~0.05° [18]. Prior to EBSD observations, the samples were mechanically polished followed by electro-polishing for approximately 30–40 s using an electrolyte of 85 vol% acetic acid and 15 vol% perchloric acid, and a voltage of 22 V at about 283 K.

#### 2.3.2. Methodology for Obtaining Deformation Substructures

Utilizing EBSD inverse pole figures (IPFs), the twin volume fraction, which is defined as the total area of the deformation twins per observed area, can be quantitatively measured as a function of the tensile deformation [19]. Similarly, the corresponding GND density can be further extracted by post-processing the EBSD-IPF maps. Based on Pantleon’s approach [20], the EBSD measurements can provide the average crystallographic orientation within an individual pixel on a 2D surface map, such that the lattice curvature and Nye’s dislocation density tensor can be derived from the orientation differences between adjacent points. Ignoring the elastic strain tensor, the Nye’s tensor can be related to the curvature tensor by the following Equation:

(1) $$\alpha_{ik}\approx \delta_{jlk}g_{ij,l},$$

where *α_ik_* is the Nye’s dislocation density tensor, *δ_jlk_* is the permutation tensor, and *g_ij,l_* is the lattice curvature obtained from EBSD orientation data [21]. Nye’s tensor is also represented by the Burgers vectors and the line vector for all dislocation types:

(2) $$\alpha_{ik}=\sum_{m=1}^{N}b_{i}^{\left(m\right)}l_{j}^{\left(m\right)}\rho^{\left(m\right)},$$

where *ρ* ^(*m*)^ is the density of the individual type of dislocations, *b* ^(*m*)^ is the Burgers vector, and *l* ^(*m*)^ is the linear vector. There are 18 different dislocation types, including 12 edge dislocations and 6 screw dislocations, for FCC structures [21]. As lattice curvatures perpendicular to the surface cannot be measured in a 2D map, a unique solution cannot be obtained using the above equations. Thus, the L1 minimization procedure is used to obtain the GND density that fits the observed lattice curvatures well, based on minimizing the total dislocation energy. A detailed discussion about the GND density measuring method can be found in Refs. [22,23,24,25]. In the present paper, the GND calculation was performed on the basis of the aforementioned method via MATLAB.

## 3. Results

### 3.1. Torsional Treatment

**Figure 1:**
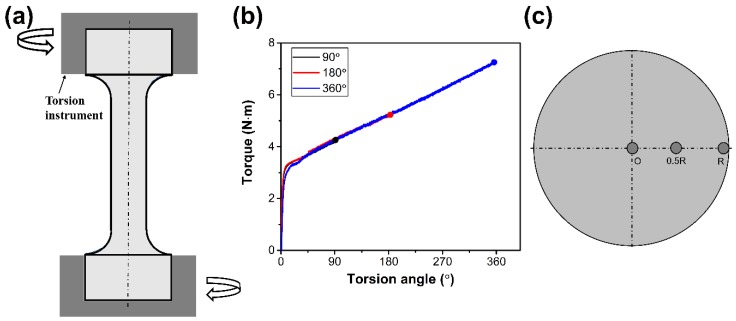
(**a**) Schematic diagram of pre-torsional deformation. (**b**) Measured torque versus torsion angle curve of Fe-22Mn-0.6C twinning-induced plasticity (TWIP) steel. (**c**) Sampling locations at the cross-section region.

In the present study, coarse-grained samples were pre-torsioned to prepare GS samples, as schematically shown in Figure 1a. This treatment was performed with torsion angles of 90°, 180°, and 360° at a nominal torsion rate of 1°/min. The representative torque versus torsion angle curves are shown in Figure 1b. For simplicity, the samples pre-torsioned to 90°, 180°, and 360°, are labeled as PT90, PT180, and PT360, respectively. For torsional deformation, the shear strain in the sample can be simply calculated as follows [14]:

(3) $$\gamma \;=\;\frac{r\theta }{l_{0}},$$

where *γ* is shear strain, *r* is the radial position from the center of the cross-section, *θ* is the torsion angle, and *l*_0_ is the initial length of the cylinder. Following Equation (3), a strain gradient is generated, that is, the shear strain along the radial direction linearly increases from the center to the surface on the cross-section of the pre-torsional cylinder. As a result, a gradient substructure is formed along the radial position.

### 3.2. Gradient Substructures After Torsional Deformation

**Figure 2:**
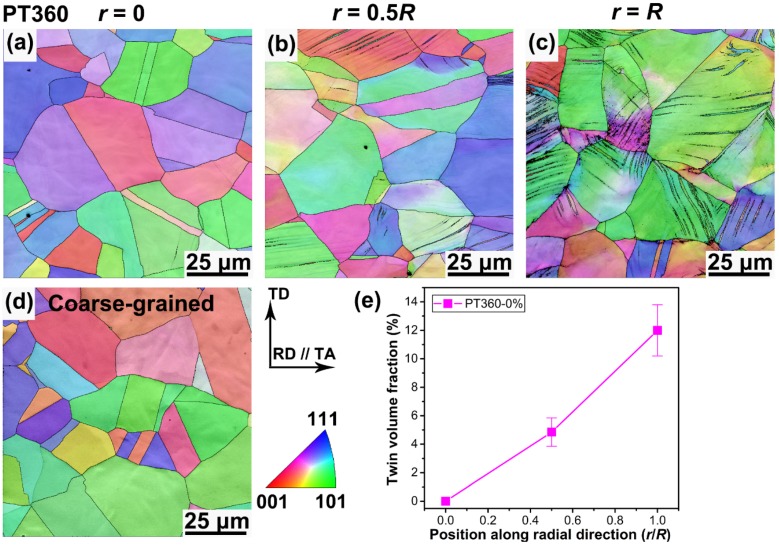
Characterization of the gradient twin substructure. Representative electron backscattered diffraction (EBSD)—inverse pole figure (IPF) maps at a position of (**a**) *r* = 0, (**b**) *r* = 0.5*R*, and (**c**) *r* = *R* on the cross-section of the PT360 sample. (**d**) Representative EBSD—IPF map for a coarse-grained sample. (**e**) Evolution of the twin volume fraction as a function of position.

To reveal the gradient substructures generated by torsional deformation, three positions along radial direction from the center to surface, viz., *r* = 0, *r* = 0.5*R*, and *r* = *R* (Figure 1c), were characterized for the PT360 sample using EBSD. As exhibited in the IPF maps of Figure 2a–c, no deformation twins are generated in the center region (*r* = 0), primary twins are active at the position of *r* = 0.5*R*, and two twin systems simultaneously appear in the outermost layer (*r* = *R*) due to an occurrence of the largest shear plastic strain. Figure 2d shows that there are no deformation twins in the coarse-grained sample. The average twin volume fraction was quantitatively obtained using the EBSD-IPF maps, as described in Figure 2e. To ensure the statistical reliability of the results, multiple maps at each position were collected to obtain the abovementioned information. The results indicate that the twin volume fraction gradually increases from 0% to 12.5% along the radial direction of the PT360 sample.

**Figure 3:**
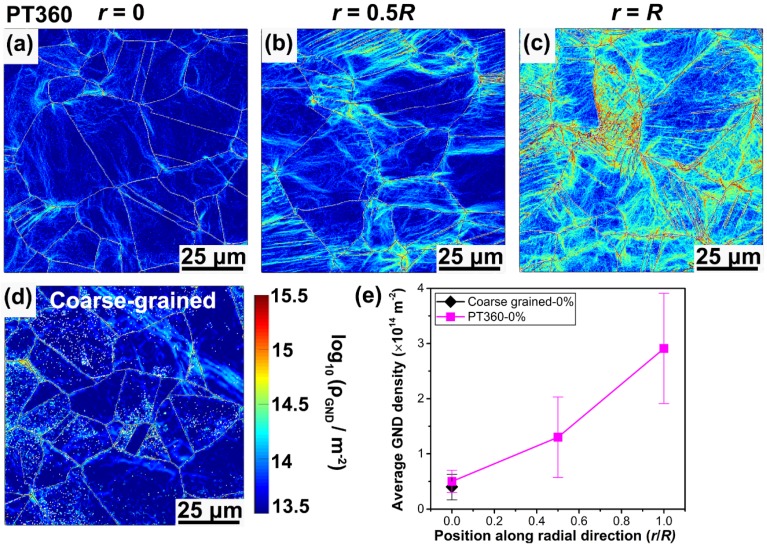
Representative EBSD-measured geometrically necessary dislocation (GND) distribution maps on the cross-section of the PT360 sample at a position of (**a**) *r* = 0, (**b**) *r* = 0.5*R*, and (**c**) *r* = *R*. (**d**) Representative GND distribution map for a coarse-grained sample. (**e**) Evolution of averaged GND density as a function of position.

Figure 3a–c shows the corresponding GND density distribution maps for the PT360 sample. The transition from blue to red color in these maps represents a change of the GND density from 13.5 to 15.5 in log_10_ scale of lines per square meter. The amount of bright (red and yellow) regions increases from the center to the surface of the cross-section, indicating a gradual increase of the GND density along the radial direction across the sample-scale. In addition, the color distribution is non-homogeneous in each map, which reflects that the GND densities are non-homogeneously distributed within the grains, and are primarily concentrated at/near grain boundaries and twin boundaries. Figure 3d presents the corresponding GND density map in the processed coarse-grained sample. Figure 3e shows the average value (*ρ_ave_*) of GND density, which is 0.5 × 10^14^ m^−2^, 1.3 × 10^14^ m^−2^, and 3.0 × 10^14^ m^−2^ at a position of *r* = 0, 0.5*R*, and *R* for the PT360 sample, respectively, and 0.4 × 10^14^ m^−2^ for the coarse-grained sample. A much higher GND density appears in the near-surface regions (*r* = *R*) due to an occurrence of the largest shear plastic strain and the formation of numerous deformation twin boundaries, which hinder the dislocation motion and locally induce an additional accumulation of GNDs. Therefore, the GS TWIP steels have a gradual increase in twin volume fraction and dislocation density along the radial direction.

### 3.3. Tensile Properties

**Figure 4:**
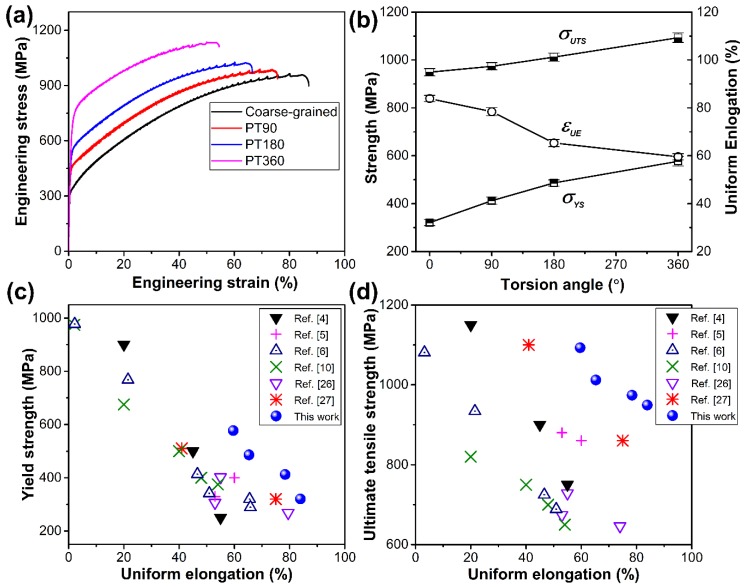
Mechanical properties of coarse grained and gradient-substructured (GS) (PT90, PT180, and PT360) samples. (**a**) Engineering stress-strain curves. (**b**) Yield strength, ultimate tensile strength, and uniform elongation as a function of the torsion angle. (**c**) Yield strength versus uniform elongation. (**d**) Ultimate tensile strength versus uniform elongation of pre-torsional samples compared to other TWIP steel samples reported in the references.

Figure 4a presents the tensile engineering stress-strain curves of coarse-grained and GS (PT90, PT180, and PT360) samples. Some specific tensile properties as a function of torsion angle are summarized in Figure 4b. Obviously, pre-torsional processing can significantly improve the yield strength (*σ_ys_*) and ultimate tensile strength (*σ_uts_*) of the present Fe-22Mn-0.6C TWIP steel, while retaining the remarkable ductility. The *σ_ys_* increases from 321 MPa for the coarse-grained sample to 413, 487, and 577 MPa for the PT90, PT180, and PT360 samples, respectively. Meanwhile, the *σ_uts_* increases from 949 MPa for the coarse-grained sample to 974, 1012, and 1093 MPa for the PT90, PT180, and PT360 samples, respectively. Particularly, the PT360 sample still retains a high uniform elongation (*ε_ue_*), viz., 60%. The tensile properties achieved in this research are also compared with other TWIP steels reported in Refs. [4,5,6,10,26,27], as indicated in Figure 4c,d. The comparisons among them indicate a superior combination of yield strength, ultimate tensile strength, and tensile ductility in the present TWIP steels with gradient twin and dislocation substructures. For example, Kang et al. [4] prepared a precipitation-containing Fe-18Mn-0.6C-1.5 Al TWIP steel, but the *ε_ue_* (45%), *σ_ys_* (500 MPa), and *σ_uts_* (900 MPa) are about 25%, 13%, and 18% lower than those of the PT360 sample, respectively. Kalsar et al. [11] produced a cold rolled Fe-10Mn-0.5C-3Ni TWIP steel with *σ_ys_* of 550 MPa, which is comparable to that of the PT360, but its *ε_ue_* is 10%, which is 83% lower than that of the PT360 sample. As a result, it is reasonable to note that the GS design can be reasonably used to enhance strength with a moderate loss of ductility, providing a synergy between strength and ductility.

### 3.4. Substructural Evolution During Tension

**Figure 5:**
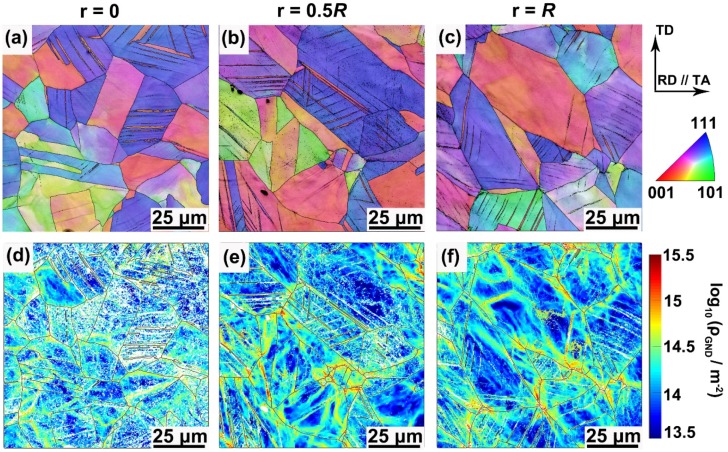
(**a**–**c**) Representative EBSD-IPF and (**d**–**f**) corresponding GND density distribution maps at an engineering strain of 20% for the coarse-grained sample.

**Figure 6:**
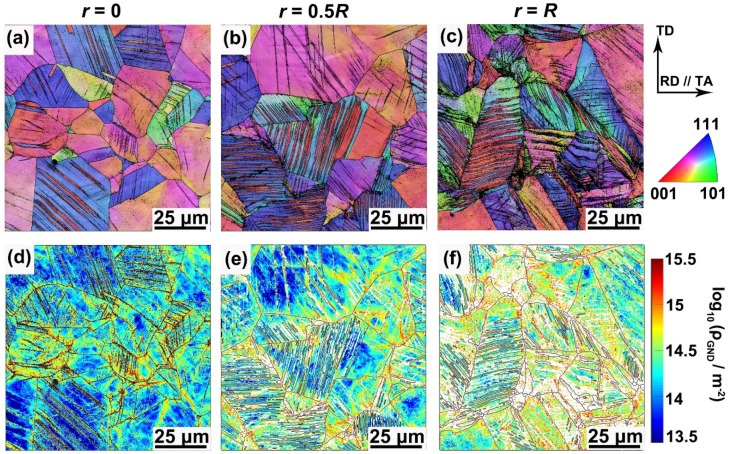
(**a**–**c**) Representative EBSD-IPF and (**d**–**f**) corresponding GND density distribution maps at an engineering strain of 20% for the PT360 sample.

In order to understand the relations between mechanical properties and underlying deformation mechanism of the GS TWIP steel samples, the cross-sectional microstructures along the radial direction (*r* = 0, 0.5*R*, *R*) of the coarse-grained and PT360 samples after tensile deformation were characterized and compared here. Figure 5 shows substructure distribution maps of the coarse-grained sample tensioned to an engineering strain of 20%. It is clearly seen that twin and dislocation substructures were generated in the deformed coarse-grained sample. The grain boundaries and twin boundaries hinder the dislocation motion, leading to pile-ups of high GNDs. Compared with Figure 2d and Figure 3d, one can see that the twin volume fraction and the overall GND density increase obviously. Additionally, there are no obvious differences in substructures among different positions in the deformed coarse-grained sample. Figure 6 presents twin and GND density distribution maps along the radial direction of the 20% tensioned PT360 sample. Similar to the prepared PT360 sample (Figure 2a–c and Figure 3a–c), gradient substructures continue to exist in the tensioned PT360 sample, but much more deformation twins and GNDs appear after tensile deformation from a qualitative view.

**Figure 7:**
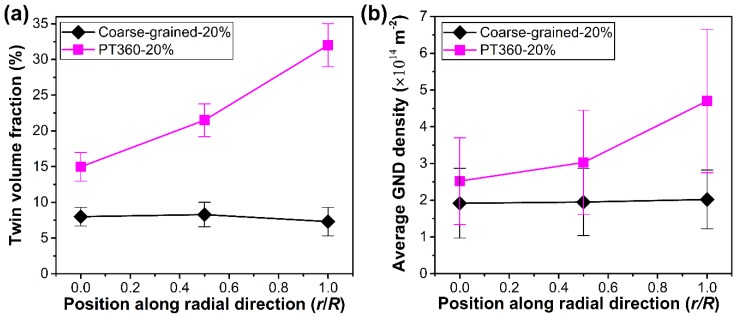
Evolution of (**a**) average twin volume fractions and (**b**) averaged GND densities as a function of positions for coarse-grained and PT360 samples pre-strained to 20% engineering strain.

The average twin volume fraction and GND density along the radial direction of two deformed samples were quantitatively extracted from the above maps (Figure 5 and Figure 6). Figure 7a shows that at 20% engineering strain, the twin volume fraction at positions of *r* = 0, 0.5*R*, and *R* of the PT360 sample is approximately 2, 2.9, and 4.3 times that of the coarse-grained sample, respectively. Figure 7b shows that the average GND density at positions of *r* = 0, 0.5*R*, and *R* of the PT360 sample is about 1.3, 1.5, 2.5 times that of the coarse-grained sample, respectively. Obviously, there is higher twin volume fraction and GND density in the bulk of the pre-tensioned PT360 TWIP steels. At positions of 0.5*R* and *R*, the higher substructure density in the PT360 sample can be attributed to a strain-hardened shell generated by the pre-torsional treatment. Within the center region (*r* = 0), the higher GND density and twin volume fraction of the PT360 sample during deformation are rationalized by two reasons. Due to variation in elastic constants and yield strength among grains, large amounts of deformation twins and GNDs are generated to achieve strain continuity across the grains in the coarse-grained and gradient samples [28]. In this case, these high GNDs are mainly blocked by grain boundaries and by newly generated twin boundaries during the tensile deformation (Figure 5 and Figure 6). On the other side, in contrast to the coarse-grained samples, extra GNDs, and deformation twins are necessarily generated at the sample-scale to accommodate the large strain partitioning and strain gradients due to mechanical incompatibility between the hard shell and soft core of the PT360 sample during tensile deformation. These can explain why substructure densities in the center region are initially nearly the same in the undeformed coarse-grained and PT360 samples (Figure 2a,d and Figure 3a,d), but after tensile deformation, higher substructure densities appear in the center region of the PT360 sample compared to that of the coarse-grained sample (Figure 5a,d and Figure 6a,d).

## 4. Discussion

### 4.1. Dislocation Strengthening

According to Ashby’s theory, the total dislocation density is the sum of the GND density and statistically stored dislocation (SSD) density [29]. The flow stress due to dislocation strengthening (*σ_dislocation_*) can be described by Taylor’s Equation as follows [30]:

(4) $$\sigma_{dislocation}=MaGb\sqrt{\left(\rho_{G}+\rho_{S}\right)},$$

where *M* is the Taylor factor, *G* is the shear elastic modulus, *b* is the magnitude of the Burger’s vector, α is the short-range interaction coefficient between dislocations, *ρ_G_* is the GND density, and *ρ_S_* is the SSD density. Based on some geometrical assumptions, Ashby believed that the GND density increased with reducing the dislocation mean free path (MFP) and increasing the shear strain in materials, while the SSD density was nearly proportional to the shear strain squared [29]. In the present TWIP steel, the dislocation MFP depends on grain size and twin volume fraction. Thus, more GNDs (Figure 3e) can be generated near the surface region of the pre-torsioned GS TWIP steel due to combined effects of larger torsional strain (Equation (3)) and more deformation twins (Figure 2e). After 20% tensile strain, there are much more deformation twins and GNDs in the GS TWIP steel than in the coarse-grained TWIP steel (Figure 7). In addition to the extra GNDs, a higher density of SSD should also form in the GS TWIP steel [17,29]. Following Equation (4), higher dislocation strengthening causes higher yield strength and tensile strength.

### 4.2. Back Stress Induced Strengthening and Hardening

**Figure 8:**
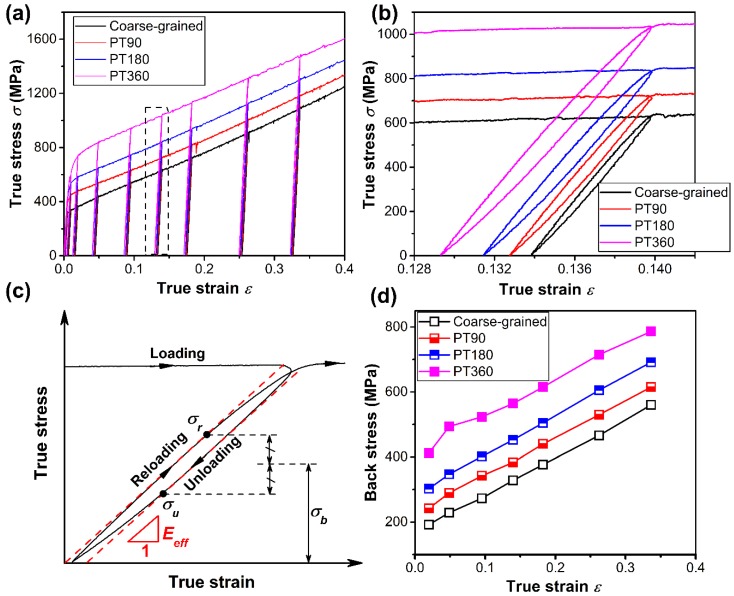
Back stress of coarse-grained and GS TWIP steel samples. (**a**) Loading-unloading-reloading (LUR) true stress-strain curves. (**b**) Enlarged view of typical hysteresis loops marked by a black dashed box in (**a**). (**c**) Schematic diagram illustrating the characteristics of hysteresis loops. (**d**) Variation of measured back stress with true strain.

GNDs not only contribute to dislocation strengthening as individual obstacles, but also generate a long-range back stress, which impedes dislocation emission from the dislocation sources [31,32]. In other words, higher flow stress is needed to overcome this field to sustain further plastic deformation. Several studies have reported that high back stress induces strengthening and hardening in heterogeneous-structured materials [8,33,34,35,36]. To measure the back stress generated during tensile deformation, LUR tests were performed on the GS (PT90, PT180, PT360) and coarse-grained samples. Each specimen was repeatedly loaded and unloaded at engineering strain levels of 2%, 5%, 10%, 15%, 20%, 30%, and 40% (corresponding true strains of 0.02, 0.05, 0.095, 0.14, 0.18, 0.26, 0.33), as exhibited in LUR tensile testing curves (Figure 8a). Figure 8b shows a typical LUR loop at 0.14 true strain for each specimen. The hysteresis loop is evidence of the occurrence of a reverse yield when the overall applied stress is still in tension during unloading. As schematically depicted in Figure 8c, the back stress (*σ_b_*) can be calculated following the method proposed by Yang et al. [37]:

(5) $$\sigma_{b}=\frac{\sigma_{r}+\sigma_{u}}{2},$$

where *σ_r_* and *σ_u_* is the reloading yield stress and unloading yield stress, respectively. The *σ_r_* and *σ_u_* in each hysteresis loop can be determined by the point at which the effective elastic modulus (*E_eff_*) is reduced by 10%, which was also adopted in some recent studies [8,34]. Figure 8d exhibits the back stress measured using the above method with increasing true strain for the coarse-grained and GS (PT90, PT180, PT360) samples. The back stress increases as the strain increases for all samples, indicating that GNDs continuously accumulate during deformation. For comparison, the GS sample possesses much higher back stress than the coarse-grained sample at all studied strain ranges. The back stress induced hardening can be regarded as the slope of the curve in Figure 8d. Particularly, the PT360 sample possesses at least the double back stress of the coarse-grained sample prior to a true strain of 0.05, and thereafter possesses nearly 60% higher back stress than the coarse-grained sample. Such a high back stress is in accord with pile-ups of more GNDs in the PT360 samples (Figure 7). As a result, the back stress strengthening and induced strain hardening further improved the flow stress and retained tensile ductility of the GS TWIP steel samples.

## 5. Conclusions

In the present study, torsional treatment was used to generate gradient substructures with the aim of improving the yield strength and retaining the tensile ductility of TWIP steels. The main conclusions are drawn as follows:

(1) Torsional treatment induced gradient deformation twin and dislocation substructures in the TWIP steel. The twin and dislocation densities gradually increased along the radial direction from center to the surface of the GS TWIP steel sample;
(2) Tensile tests of the GS TWIP steel samples showed large improvements in the yield strength and ultimate tensile strength with only a modest compromise to the tensile ductility;
(3) Microstructural observations revealed that the gradient substructures still remained during tensile deformation, leading to a strain incompatibility and strain partitioning between the surface and center of the tensioned sample. As a result, more twins and GNDs were formed in the center region (*r* = 0) of the GS sample than in the bulk of the coarse-grained sample during tension;
(4) LUR tests showed that higher back stress was generated in the GS samples, which originated from the strain incompatibility between the surface and center across the sample-scale together with GND accumulation at/near grain and twin boundaries at the grain-scale;
(5) Consequently, the high yield and ultimate tensile strengths originated from a synergetic effect of dislocation strengthening and gradient substructure-induced back stress strengthening. Additionally, the high back stress induced high strain hardening, further leading to large tensile ductility of the GS TWIP steels.
